# High Isolation MIMO Antenna System for 5G N77/N78/N79 Bands

**DOI:** 10.3390/mi15060721

**Published:** 2024-05-29

**Authors:** Xuanhe Wei, Jiaping Lu, Youming Miao, Jianlin Huang, Zhizhou Chen, Gui Liu

**Affiliations:** College of Electrical and Electronic Engineering, Wenzhou University, Wenzhou 325035, China; 21451841026@stu.wzu.edu.cn (X.W.); 22451841024@stu.wzu.edu.cn (J.L.); 22461237086@stu.wzu.edu.cn (Y.M.); 194511981414@stu.wzu.edu.cn (J.H.); 20170157@wzu.edu.cn (Z.C.)

**Keywords:** 5G new radio (5G NR), sub-6 GHz, MIMO, dual-band antenna, metal-frame smartphone

## Abstract

This paper presents a symmetric dual-band multiple-input multiple-output (MIMO) antenna system tailored for fifth-generation (5G) mobile terminals. Operating within the 5G frequency bands N77/N78 (3.4–3.6 GHz) and N79 (4.8–5.0 GHz), the proposed MIMO system achieves high isolation between adjacent antenna elements through slotting and self-decoupling technologies. Antenna elements are strategically positioned on two frames perpendicular to the smartphone’s main board. Each antenna element integrates a rectangular microstrip radiator on the inner frame surface, accompanied by a grounded rectangular ring on the outer frame surface. The feed line, situated atop the main board, connects to an external SMA connector located at the main board’s bottom. Measurement results reveal isolations exceeding 20 dB for the lower band and 24 dB for the higher band. The fabricated and tested MIMO antenna system demonstrates excellent agreement between simulation and measurement outcomes.

## 1. Introduction

5G networks offer higher data rates and lower latency compared to previous generations, enabling faster and more reliable wireless communication. In different countries and regions, the 5G mobile wireless standard has been assigned distinct frequency bands. For example, four telecommunications operators, namely China Mobile, China Telecom, China Unicom, and China Broadnet offer 5G services within Mainland China. The frequency band n77 (3.3–3.4 GHz) is jointly used by China Telecom, China Unicom, and China Broadcasting for 5G indoor coverage. The n78 band (3.5–3.6 GHz) is utilized by China Unicom and China Telecom. The n79 frequency band (4.8–4.9 GHz) is assigned to China Mobile. The widespread adoption of 5G mobile communication systems has heightened the importance of antenna design for 5G mobile devices. MIMO technology enhances wireless communication capacity and quality by utilizing spatial diversity and multiplexing to establish parallel data streams, improving efficiency and reliability [1,2,3]. The utilization of MIMO antennas in 5G smartphones enhances communication efficiency and elevates data transmission rates.

The design and implementation of MIMO antennas for 5G smartphones encounter numerous challenges, encompassing the need for larger radiation cell sizes, achieving antenna radiation omnidirectionality, and addressing efficiency issues. These antennas are often equipped with multiple ports to accommodate the spatial division and multiplexing requirements of MIMO systems, aiming to achieve higher gain and improved omnidirectionality [4,5]. However, this approach presents challenges in managing isolation between neighboring antenna elements within limited space, potentially leading to mutual interference in MIMO mobile phones [6]. To address these challenges, efforts can be directed toward minimizing outward radiation from antenna elements and integrating various isolation structures to prevent radiation coupling [7]. Researchers have explored numerous approaches to address the isolation challenge. For instance, [8] proposes a self-decoupled antenna pair where adjacent elements share a common ground branch, enhancing isolation. In [9], isolation performance is improved using an M-shaped slot between antenna elements. Other techniques include neutralization or offset methods, blocking techniques, orthogonal polarization strategies, and optimized antenna layouts [10,11]. Notably, recent research [12] provides a comprehensive review of decoupling methods, including external decoupling structures, orthogonal modes, and mitigation of ground effects, while also discussing emerging trends in MIMO array development for 5G smartphones.

The trend towards miniaturization and ultra-thin design in smartphones has spurred demand for compact antenna designs. Miniaturizing MIMO antennas for mobile devices presents challenges due to limited available space, making it difficult to integrate multiple elements [13]. As communication technology advances, the trend toward ultra-thin smartphones necessitates antenna miniaturization. Proposed solutions include miniaturized MIMO antennas for 5G smartphones, such as inverted loop antennas [14,15]. Ref. [16] introduces a hybrid antenna design incorporating a comb-shaped antenna, enabling frame height reduction to 6 mm or below, aligning with modern mobile device design requirements. Another trend for mobile devices is the need to support a wide range of frequency bands to achieve optimal wireless communication instead of single-band designs [17,18]. Antenna miniaturization often involves multiplexing multiple frequency bands. Techniques such as metamaterial-based designs and reconfigurable antennas have been proposed to address these challenges [13,17].

In this study, we propose a miniaturization dual-band eight-port MIMO antenna system suitable for 5G applications. Positioned on the external surface of a smartphone’s side and oriented perpendicular to the substrate to enhance isolation, the antenna design incorporates a T-shaped slot to reduce coupling between adjacent antenna elements. This configuration optimizes the current path using an offset method, resulting in a significant enhancement in isolation measured smaller than −20 dB. The ground plane is connected to the center of the lateral substrate, and the antenna elements are evenly distributed. The final dimensions of the antenna system measure 150 mm × 75 mm × 5 mm. The utilization of multi-band technology can effectively address the antenna design challenges arising from spatial constraints. The MIMO system operated at 3.4–3.6 GHz and 4.7–5.3 GHz, which can cover the 3.4–3.6 GHz and 4.8–5.0 GHz bands, authorized by the Ministry of Industry and Information Technology of China for 5G n77/n78/n79 applications. Simulation results align closely with measured data, validating the reliability of this antenna configuration.

## 2. Structure of the Proposed MIMO Antenna

**Figure 1:**
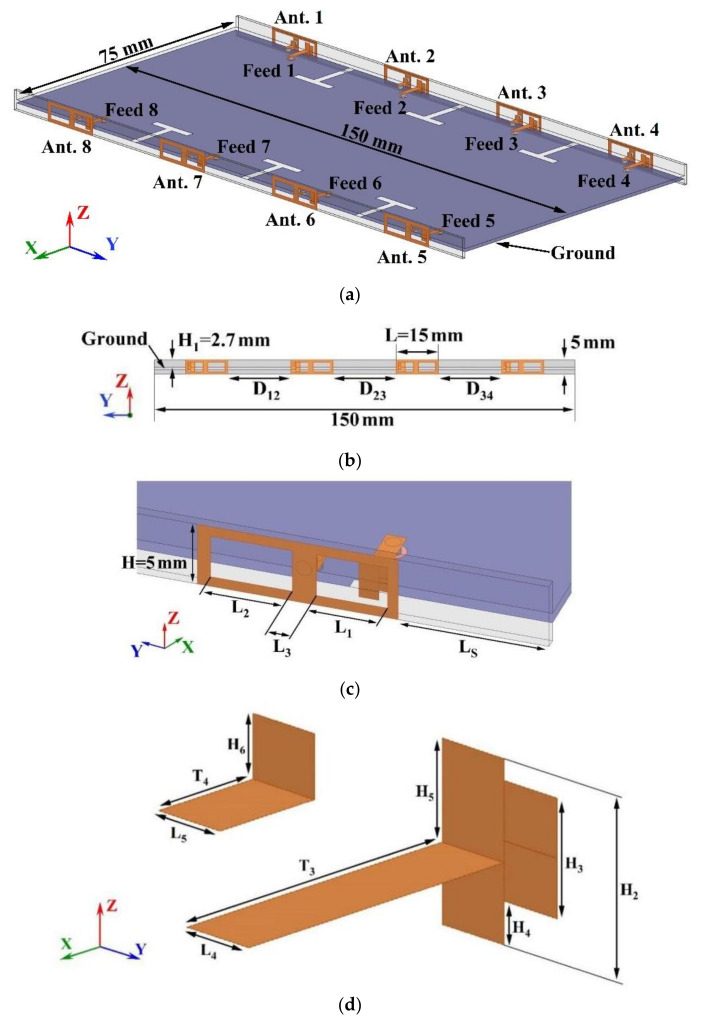
The geometry of the proposed eight-port MIMO antenna system: (**a**) Perspective view of the system; (**b**) Side view of the side dielectric substrate; (**c**) Perspective view of antenna element; and (**d**) The radiator and feed line of the antenna element.

The structure and dimensions of the proposed eight-port MIMO antenna system for the mobile terminal are shown in Figure 1. Two side dielectric substrates are perpendicular to the main dielectric substrate. Eight antenna elements are printed on both sides of the two side dielectric substrates, and the system ground plane is printed on the bottom of the main dielectric substrate. The size of the main dielectric substrate and the side dielectric substrate is 150 mm × 75 mm × 0.8 mm, and 150 mm × 5 mm × 0.8 mm, respectively. The substrate material is FR4 with a dielectric constant of 4.4 and dielectric loss tanδ of 0.02.

**Figure 2:**
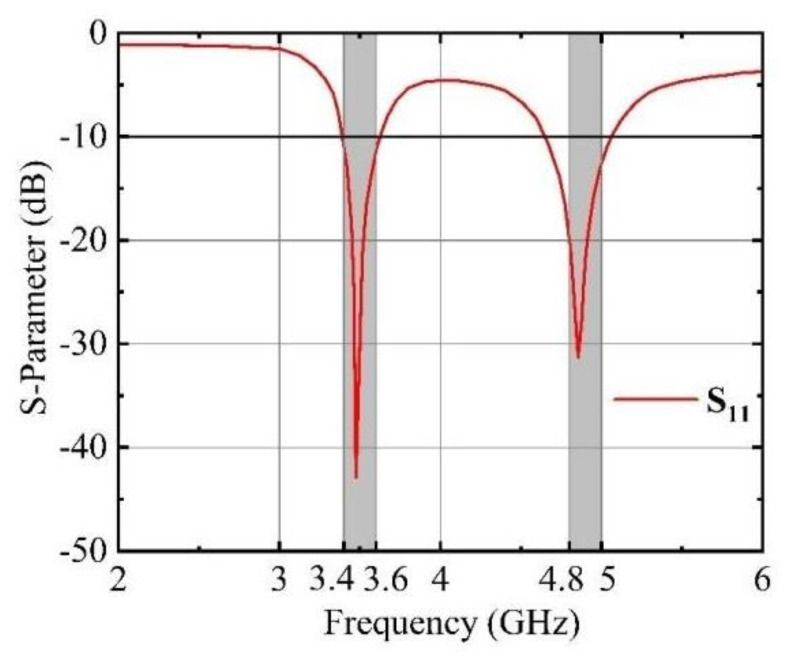
Simulated reflection coefficients of the antenna element.

Figure 1b shows the side view of the side dielectric substrate. The distance between the top of the side dielectric substrates and the upper surface of the main dielectric substrate is 2.7 mm. The distances between Antenna 1 and Antenna 2, Antenna 2 and Antenna 3, and Antenna 3 and Antenna 4 are labeled as D_12_, D_23_, and D_34_, respectively. All of these distances are set at 23 mm, meaning D_12_ equals D_23_ equals D_34_. The perspective view of the antenna element is illustrated in Figure 1c. The outer surface of the side dielectric substrate features a double-rectangular ring patch, which is connected to a rectangular patch on the inner surface of the same substrate by a via. This rectangular patch, in turn, connects to the ground plane through a via on the top surface of the main dielectric substrate. On the inner surface of the side dielectric substrate, a T-shaped patch is fed by a 50 Ω feed line originating from the top surface of the main dielectric substrate. This feed line is connected to an external SMA connector via another via. A T-shaped slot is cut between every two adjacent elements to improve the isolation between antenna elements. The dimensions of a single antenna element measure 15 mm × 5 mm × 0.8 mm. The antenna element is designed to maintain an S_11_ value of less than −10 dB within the operational frequency band. The simulation software for the antenna design is HFSS 15 (high-frequency structure simulation). The antenna element operates in two frequency bands of 3.4–3.6 GHz and 4.8–5.0 GHz. Figure 2 illustrates the S-parameters of the antenna element.

**Figure 3:**
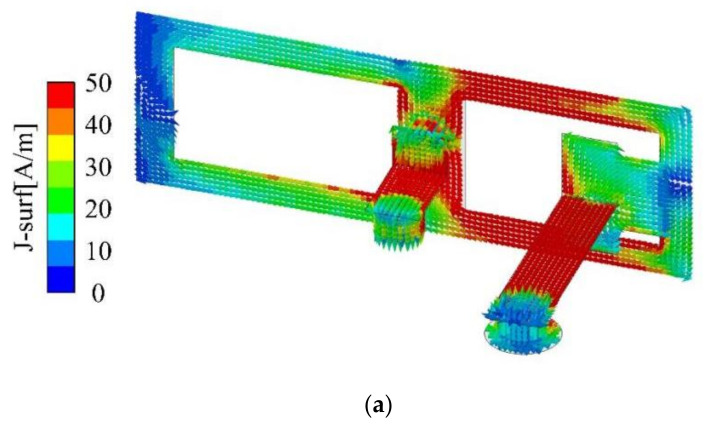

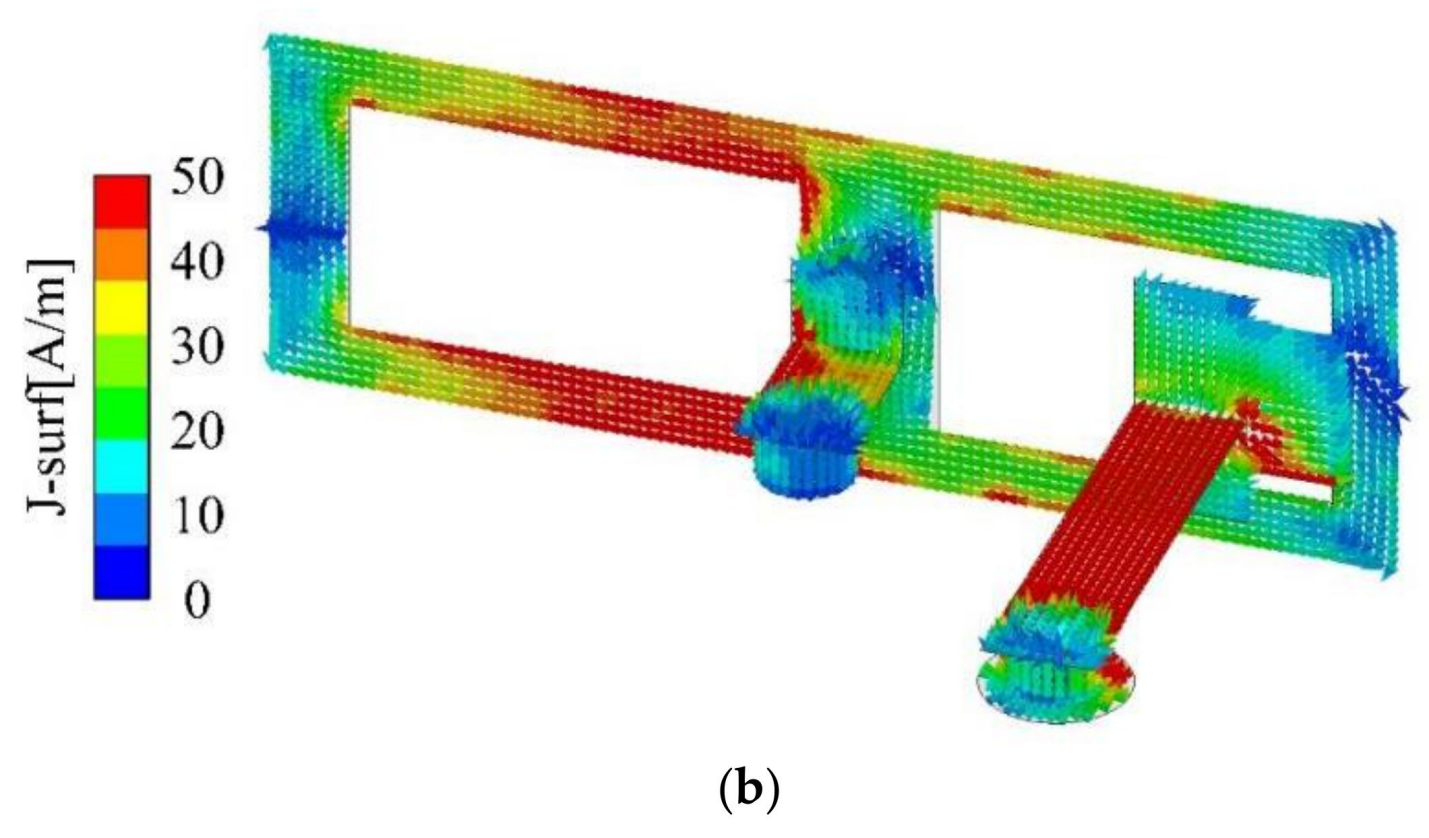
Simulated current distributions of Ant. 1 at (**a**) 3.5 GHz and (**b**) 4.9 GHz.

To elucidate the operational principle of the proposed MIMO antenna, a single antenna element is chosen for analysis. Figure 3 presents the current distribution for two frequency points in two operating frequency bands. 

The current path at 3.5 GHz, as illustrated in Figure 3a, is primarily concentrated on the central strip and the upper and lower strips on the right side of the rectangular ring patch. This corresponds to a quarter-wavelength resonant mode, which is roughly 20.4 mm in length, equating to about 0.235 times the wavelength (λ_0_). Figure 3b depicts the current distribution at 4.9 GHz, with the current predominantly focused on the upper and lower strips on the left side of the rectangular ring patch. Similarly, the current path at this frequency contributes to a quarter-wavelength resonant mode, which is approximately 16 mm, or roughly 0.26 times the wavelength (λ_0_). 

The lower operating band of the MIMO antenna is realized by the outermost rectangular structure of the antenna element. The higher frequency band is realized by the rectangular ring near the feed point, and the rectangular structure on the outside surface also has a certain positive effect on its operation.

**Figure 4:**
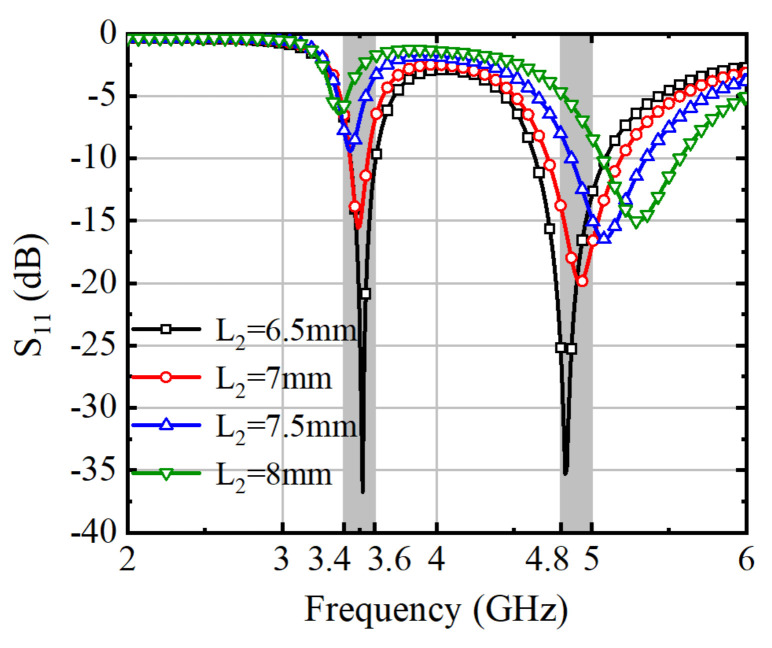
Simulated S_11_ of Ant. 1 with varying lengths of L_2_.

**Figure 5:**
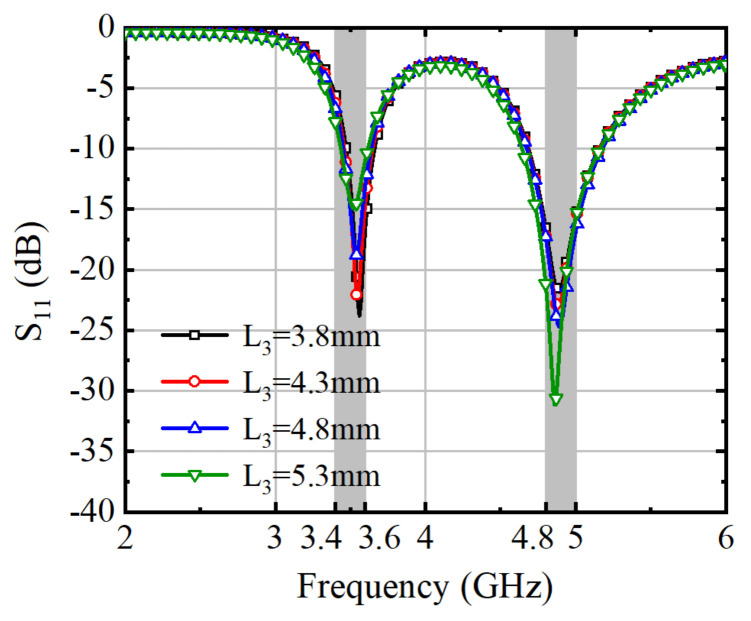
Simulated S_11_ of Ant. 1 with varying lengths of L_3_.

The S-parameters of the antenna element were analyzed and simulated with different values of L_2_ and L_3_, as shown in Figure 4 and Figure 5. Adjusting the length of L_2_ results in different current paths, which in turn affect the operating frequency. The precise impacts of these adjustments are detailed in Figure 4. Increasing the value of L_2_ leads to a shift of the two operating frequencies towards higher and lower frequencies, respectively.

The influence of L_3_ on the antenna element’s performance is shown in Figure 5. As the length of L_3_ increases towards the feed patch, it affects the two operational frequency bands of the antenna element. The size of L_3_ can be used to effectively adjust the balance of the S-Parameters for both the lower and higher frequencies. The data in Figure 5 suggest that the antenna element’s impedance can be precisely matched to accommodate different scenarios.

## 3. Antenna Analysis and Parameter Setting

**Figure 6:**
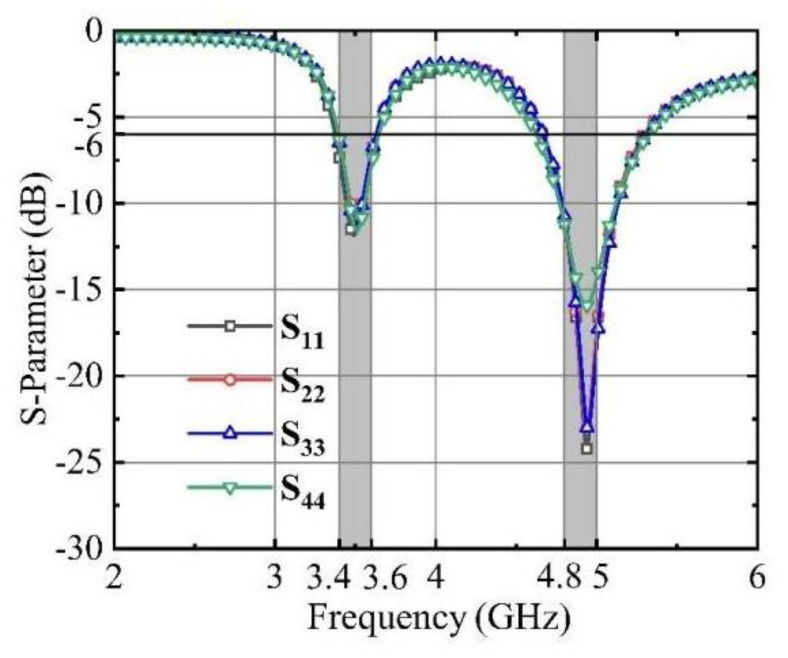
Simulated reflection coefficients of the antenna system.

Due to the inherent symmetry within the MIMO antenna system, only the pertinent S-parameters are displayed to enhance clarity and simplicity. The simulated reflection coefficients for the MIMO system are presented in Figure 6.

**Figure 7:**
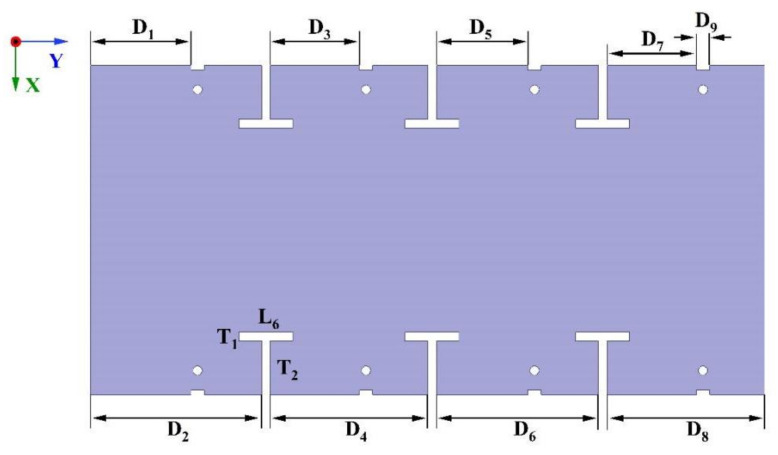
Model of the antenna system with T-shape slots on the ground.

To improve isolation within the proposed MIMO antenna system and reduce the impact of surface wave coupling, slots have been incorporated into the ground plane. These slots act as barriers to the current, effectively blocking the path that could lead to leakage and subsequent interference with other antenna elements. This technique successfully enhances isolation while avoiding the need for a larger antenna footprint or the addition of extra structural components. It offers a simple and efficient solution. As shown in Figure 7, T-shaped slots were used in the ground plane to achieve this improvement. 

**Figure 8:**
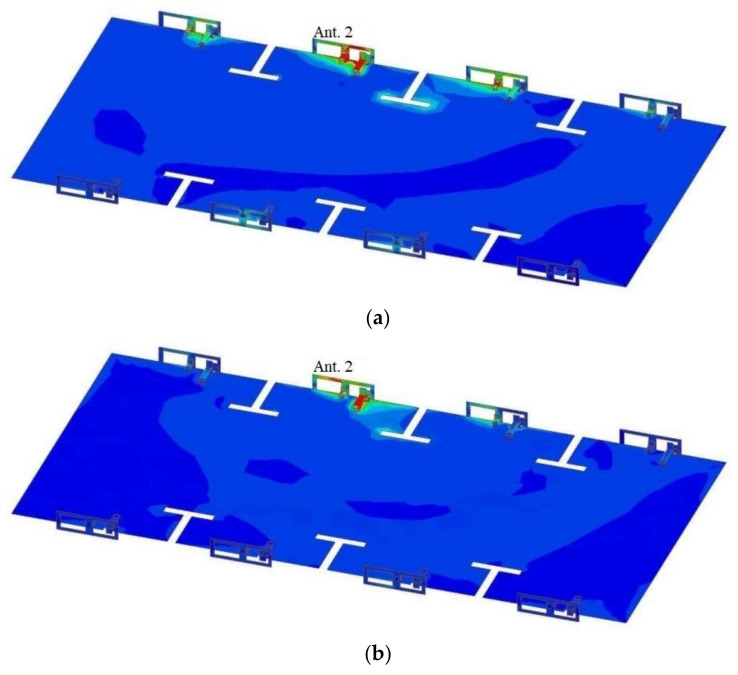
Surface current distribution of ground with T-shaped slots and antenna elements under different excitations: (**a**) Current distribution at 3.5 GHz with Antenna 2 excited; (**b**) Current distribution at 4.9 GHz with Antenna 2 excited.

These strategically placed T-shaped slots serve a specific purpose: to modify the current distribution on the ground plane, ultimately improving isolation between antennas. As depicted in Figure 8, the accumulation of surface current near these T-shaped slots impedes current flow, effectively minimizing interference among the antennas.

**Figure 9:**
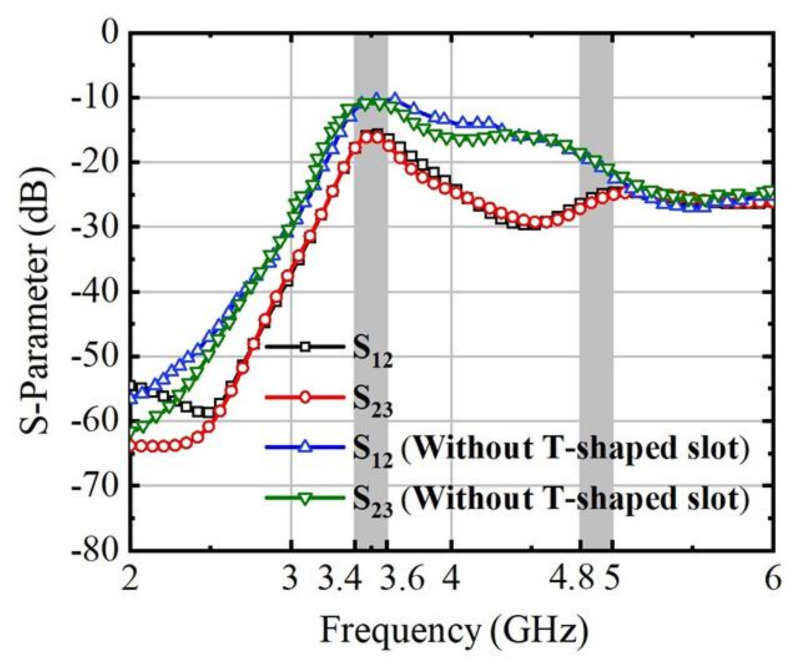
Simulated transmission coefficient of the antenna system.

The T-shaped slot, denoted as T1, possesses a width of 2 mm, while both its length (L6) and height (T2) measure 12 mm. The distance from the feed element to the boundary is fixed at 20.6 mm. Adjacent T-slots are spaced at 36 mm intervals, indicating that D2 = D4 = D6 = D8 = 36 mm. The isolation characteristics after adding the T-shaped slot of the antenna system are illustrated in Figure 9, with the peak isolation point at S_12_ from −9.7 dB to −15.7 dB.

The optimized results are presented in Table 1 below. These parameters underwent re-optimization to mitigate performance losses that arise upon integrating the antenna elements into the system.

**Figure 10:**
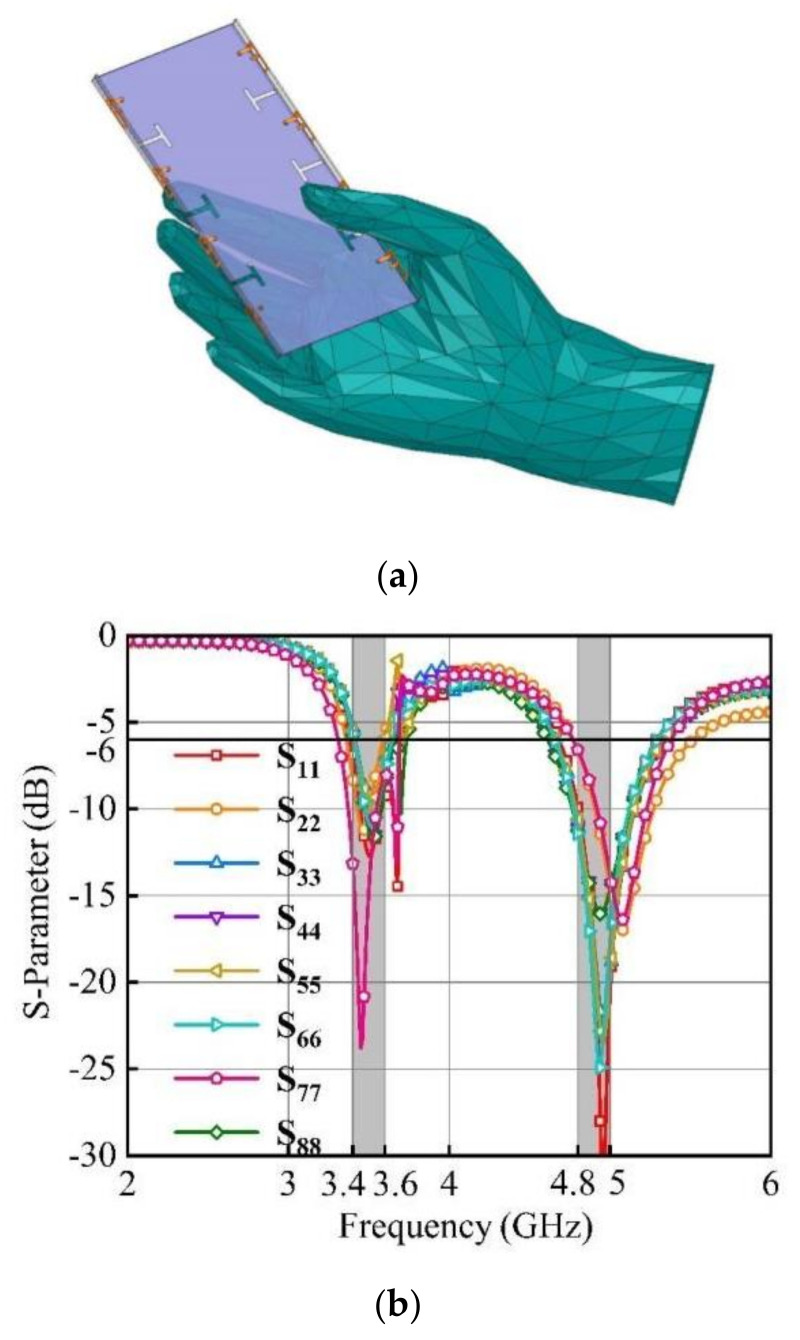
Simulated SHO mode: (**a**) Total model; (**b**) Simulated S-parameter of the antenna system.

In this paper, the single-hand operation (SHO) mode is considered. Figure 10 illustrates the simulated working state of the device when used by a user. Ant. 3, Ant. 4, Ant. 5, Ant. 6, and Ant. 7 are identified as the most significantly affected antennas. Each of these antennas demonstrates a distinct deviation of nearly 0.5 dB in their respective S-parameters.

## 4. Antenna Performance Test

**Figure 11:**
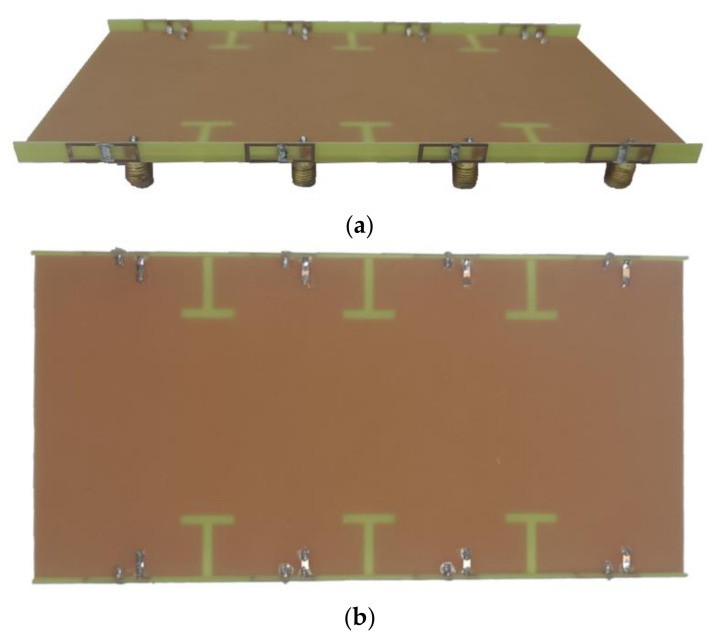
Photograph of the proposed antenna: (**a**) Perspective view; (**b**) Top view.

**Figure 12:**
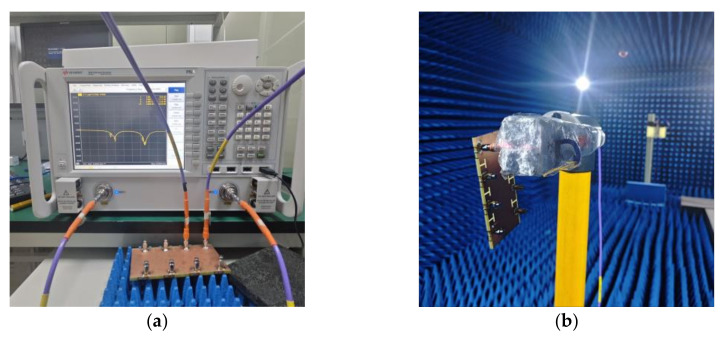
Antenna test: (**a**) S-parameter; (**b**) Far field.

To comprehensively assess the antenna performance and validate the antenna design, the antenna was fabricated and assembled, as illustrated in Figure 11. Subsequently, the actual antenna underwent welding and testing, with the test procedure outlined in Figure 12.

**Figure 13:**
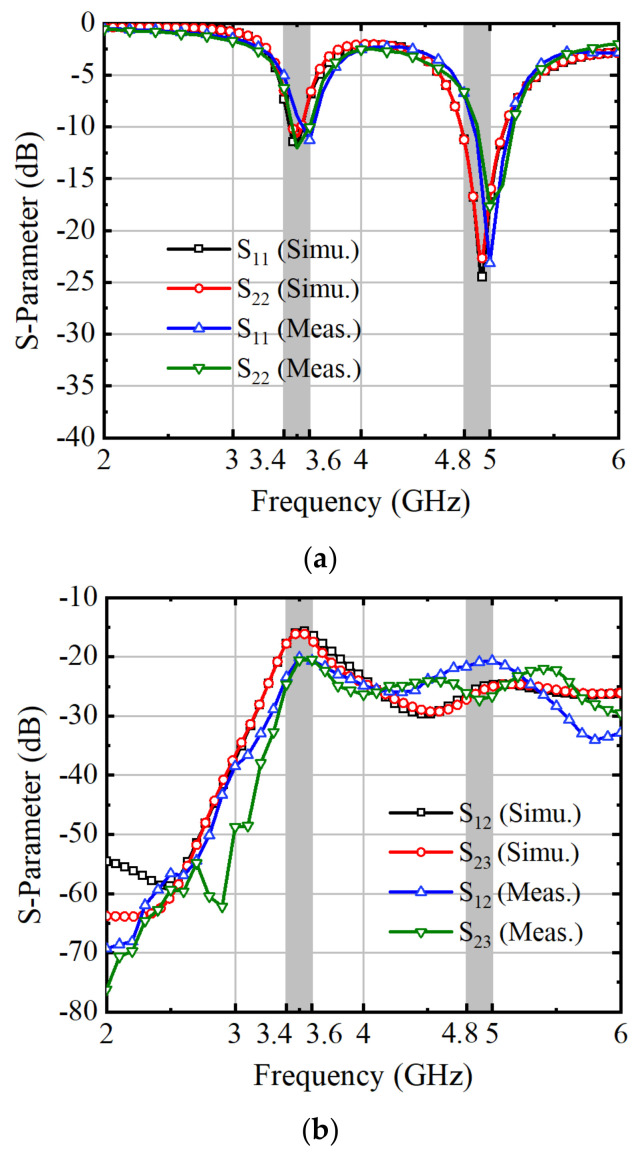
Simulated and measured S-parameters of the eight-antenna MIMO system: (**a**) Simulated and measured reflection coefficient, (**b**) Simulated and measured transmission coefficient.

The measured results are compared with the simulation results in the figure below. Add 50 Ω terminals for ports other than the test port. The antenna’s measured S-parameters show frequency deviations due to manufacturing inaccuracies and manual assembly issues. Figure 13a illustrates the proposed antenna system maintaining a −6 dB threshold within the operational frequency bands of 3.4–3.6 GHz and 4.8–4.9 GHz, ensuring its effectiveness in practical applications. Furthermore, Figure 13b demonstrates isolation levels exceeding 20 dB between different antenna elements.

**Figure 14:**
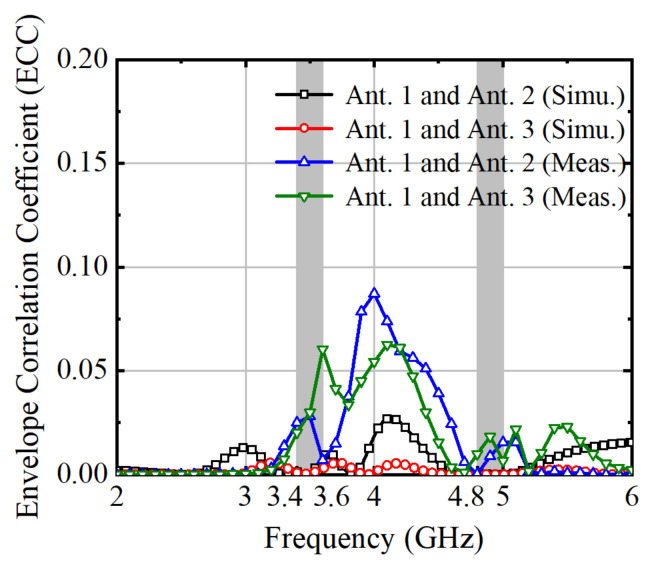
Envelope correlation coefficient of the MIMO system with uniform distribution.

The envelope correlation coefficient (ECC) stands as a cornerstone metric within multiple-input multiple-output (MIMO) systems, crucial for gauging the correlation between signals received by multiple antennas. Its significance lies in its ability to indicate signal correlation levels, directly impacting the system’s diversity performance and, consequently, its overall efficiency and reliability. In practical MIMO deployments, a low ECC value signifies reduced correlation and enhanced spatial diversity, vital for mitigating fading effects and combating interference in wireless communication channels. By leveraging ECC analysis, engineers can refine antenna designs, fine-tuning them to optimize system performance under dynamic and challenging propagation conditions. This analytical approach not only aids in maximizing data rates but also ensures robustness in real-world wireless communication scenarios. Therefore, ECC serves as a pivotal tool in the arsenal of MIMO system optimization, empowering engineers to craft resilient and efficient wireless communication solutions. The ECC can be calculated by Equation (1), and the calculated ECC is shown in Figure 14. Notably, the ECC value is below 0.066 within the desired frequency band, which falls below the threshold of 0.5 required for normal MIMO antenna operation.

(1) $$ECC=\frac{\iint_{4\pi }^{\;}A_{ij}\left(\theta ,\phi \right)\sin\left(\theta \right)d\theta d\phi }{\sqrt{\int_{4\pi }^{\;}A_{ii}\left(\theta ,\phi \right)\sin\left(\theta \right)d\Omega \int_{4\pi }^{\;}A_{ij}\left(\theta ,\phi \right)\sin\left(\theta \right)d\Omega }}$$

**Figure 15:**
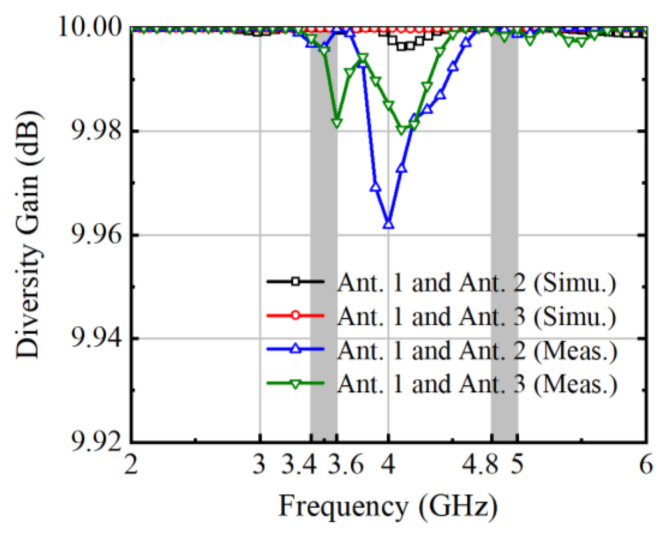
Calculated DG.

Diversity gain (DG) plays a pivotal role in assessing the efficacy of MIMO antenna systems, offering insights into their ability to improve signal reliability and robustness in challenging propagation environments. Derived from the envelope correlation coefficient (ECC) calculation, DG quantifies the system’s capacity to exploit spatial diversity, thereby mitigating the adverse effects of fading and interference. By leveraging multiple antennas strategically positioned, MIMO systems can achieve significant diversity gains, enhancing signal reception quality and overall system performance. This diversity enables the system to capitalize on multipath propagation, effectively mitigating signal fading and enhancing communication reliability. As a result, DG serves as a critical metric for evaluating and optimizing MIMO antenna designs, ensuring they deliver superior performance in real-world wireless communication scenarios. DG can be calculated by Equation (2). The computational outcomes are depicted in a graphical representation, illustrated in Figure 15, indicating values exceeding 9.97 dB across operational frequency bands.

(2) $$\mathrm{DG}=10\left(\mathrm{dB}\right)\times \sqrt{1-{\left|\mathrm{ECC}\right|}^{2}}$$

**Figure 16:**
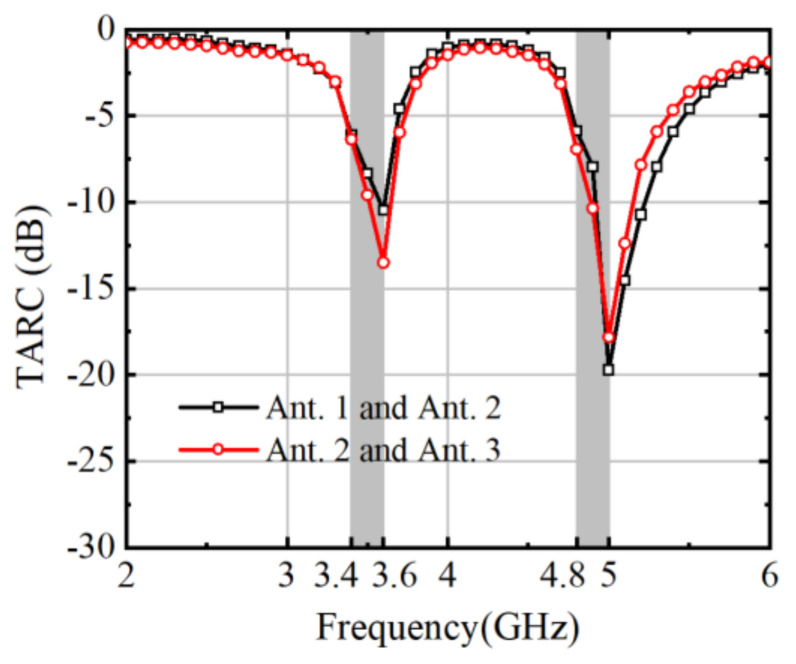
Antenna S-parameters under full operation of eight ports.

Figure 16 plays a pivotal role in illustrating the significance of the total active reflection coefficient (TRAC) in evaluating MIMO antenna system performance. TRAC, representing both magnitude and phase aspects of reflection coefficients across active ports, offers insights into impedance matching and radiation pattern characteristics. Particularly crucial in MIMO setups, TRAC analysis aids in identifying impedance mismatches and optimizing antenna designs for efficient signal transmission and reception.

**Figure 17:**
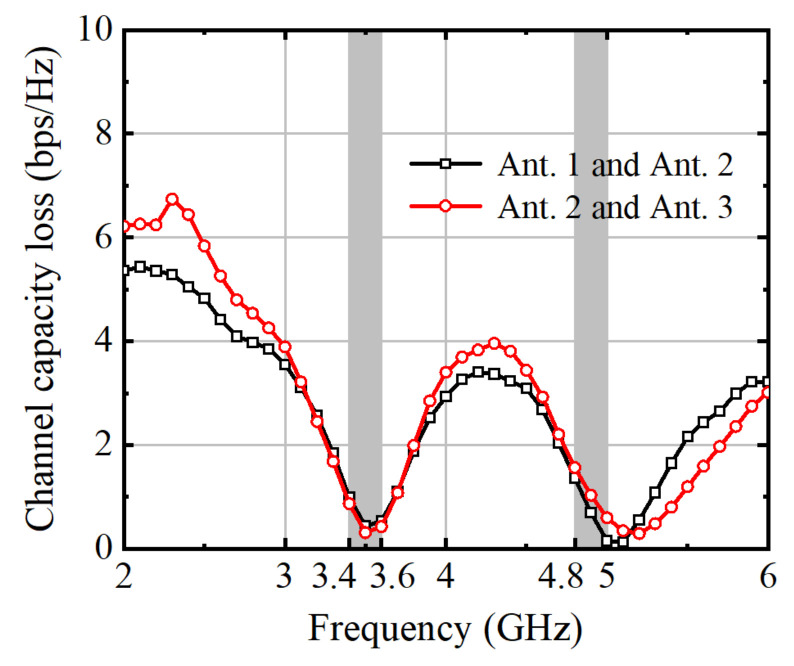
Calculated CCL.

Assessing the channel capacity loss (CCL) in MIMO antenna performance is crucial for understanding the correlation between signals received by multiple antennas. CCL quantifies the degradation in channel capacity due to correlation among received signals, which can arise from factors such as antenna spacing and environmental conditions. By evaluating CCL, engineers gain insights into the extent to which spatial diversity is effectively harnessed in the MIMO system. High CCL values indicate increased correlation and reduced diversity, potentially leading to diminished system performance in terms of data throughput and reliability. Therefore, understanding and mitigating CCL is essential for optimizing MIMO antenna configurations and maximizing the system’s capacity to exploit spatial diversity for enhanced communication performance. The CCL can be calculated by Equation (3), and shown in Figure 17. The maximum allowed CCL value for MIMO antenna elements is 0.4 bps/Hz or less. Figure 17 illustrates that the CCL value between the correlated antenna elements is below 0.33 bps/Hz at 3.5 GHz, indicating reduced losses.

(3) $$CCL=-log(\left(1-\left(mag{\left(S\left(i,i\right)\right)}^{2}+mag{\left(S\left(i,j\right)\right)}^{2}\right)\right)*(1-(mag{\left(S\left(j,j\right)\right)}^{2}\;\;\;\;\;\;\;\;\;\;+mag{\left(S\left(j,i\right)\right)}^{2})-(\left(conjg\left(S\left(j,j\right)\right)*S\left(j,i\right)+conjg\left(S\left(i,j\right)\right)*S\left(i,i\right)\right)\;\;\;*\left(conjg\left(S\left(i,i\right)\right)*S\left(i,j\right)+conjg\left(S\left(j,i\right)\right)*S\left(j,j\right)\right)))/log\left(2\right)$$

**Figure 18:**
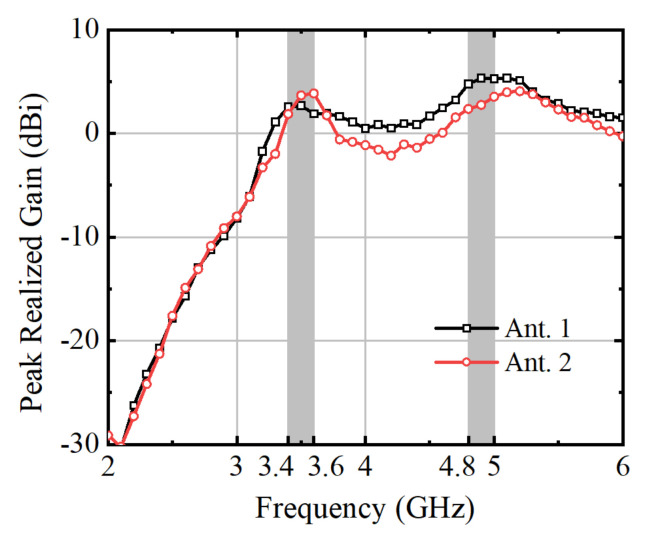
Measured antenna peak realized gain.

**Figure 19:**
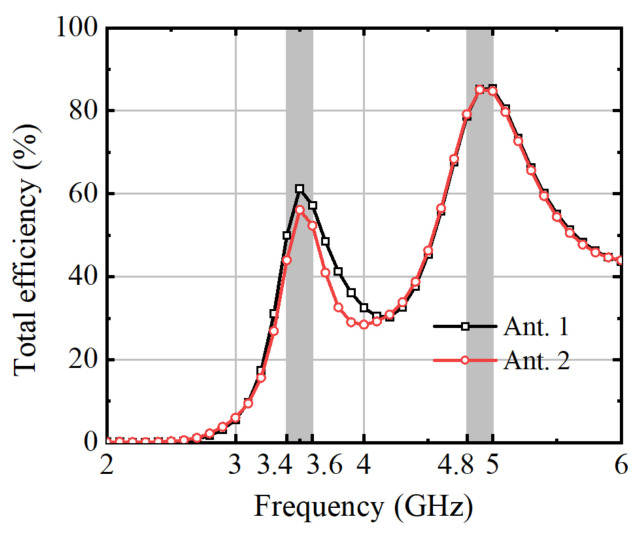
Measured total efficiency.

Figure 18 and Figure 19 showcase the frequency-dependent antenna gain and efficiency. The gain, depicting the performance of Ant. 1 in the eight-antenna MIMO system operation, reveals a value of 1.86 for the measured antenna peak realized gain. This parameter signifies the maximum gain achieved by the antenna at specific frequencies during measurement, indicating its effectiveness in capturing or transmitting signals. Notably, within the frequency bands of 4.8–5.0 GHz, the antenna gain exceeds 2.36, demonstrating its ability to amplify signals effectively. It reaches a maximum value of 2.3 at 5.0 GHz, suggesting optimal performance in signal reception or transmission at this frequency.

The gain efficiency at the 3.5 GHz band is close to 43%, indicating that approximately 43% of the input power is effectively radiated as electromagnetic energy by the antenna at this frequency. This efficiency underscores the antenna’s suitability for applications within this frequency range. Moreover, the efficiency observed at 4.8 GHz is notably higher, reaching 79%. This substantial increase in efficiency suggests enhanced performance and effectiveness of the antenna in transmitting or receiving signals at this frequency, making it well-suited for use in communication systems operating within the 4.8 GHz band.

**Figure 20:**
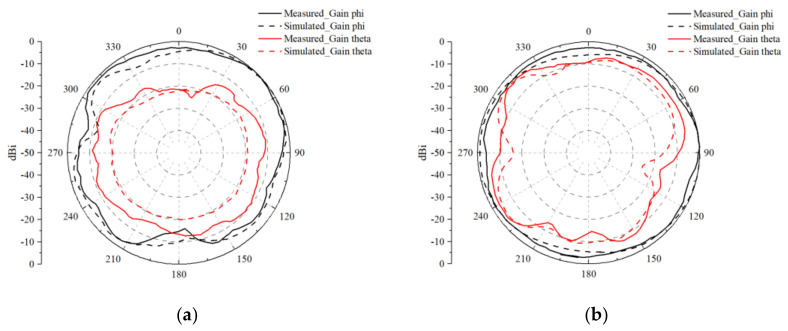
Measured 2D antenna radiation patterns at 3.5 GHz for the eight-antenna MIMO system with uniform distribution: (**a**) Ant. 1 in the xoy plane; (**b**) Ant. 1 in the yoz plane.

**Figure 21:**
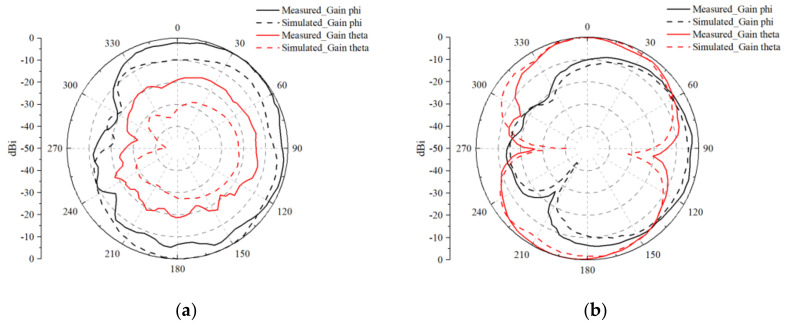
Measured 2D antenna radiation patterns at 4.9 GHz for the eight-antenna MIMO system with uniform distribution: (**a**) Ant. 1 in the xoy plane; (**b**) Ant. 1 in the yoz plane.

The 2D radiation patterns of Antenna 1 at the respective resonance frequencies are presented in Figure 20 and Figure 21. Moreover, a comparison between the 2D directional characteristics of the test and simulation results demonstrates a high level of similarity in the overall directionality. This close alignment between the measured and simulated radiation patterns validates the accuracy and reliability of the simulation model in predicting the antenna’s performance. Engineers and researchers can have confidence in using simulation tools to predict MIMO antenna behavior accurately, thus streamlining the antenna development process and reducing the need for extensive testing. The consistency between the measurement and simulated results also provides valuable insights for future MIMO antenna design iterations and optimizations, enabling engineers to leverage simulation data to explore various design parameters and configurations, aiming to further enhance antenna performance and meet specific application requirements. Overall, the close match between the 2D radiation patterns obtained from both measurement and simulation demonstrates the robustness and reliability of the proposed MIMO antenna design, paving the way for its successful integration into diverse wireless communication systems and applications.

For a detailed insight into the specific operational performance, pertinent antenna details are summarized in Table 2.

Table 2 provides essential antenna details for a comprehensive understanding of its operational performance, including operating band, isolation, efficiency, ECC, and size. These specifications offer valuable insights into the antenna’s capabilities under various operating conditions, aiding engineers in assessing its suitability for specific applications and making informed design decisions. As can be observed from Table 2, the proposed dual-band MIMO antenna shows high isolation, high efficiency, and small ECC.

## 5. Conclusions

The proposed eight-antenna MIMO system, designed for integration into a mobile terminal for 5G prototype validation, exhibits promising performance characteristics. With dimensions of 150 mm × 75 mm × 5 mm, the antenna system effectively covers the frequency bands of 3.4–3.6 GHz and 4.8–5.0 GHz. Each antenna element features a dual rectangular ring structure, contributing to a reduction in overall thickness while maintaining robust functionality. The measured isolation, surpassing 20 dB, ensures minimal interference between antenna elements, enhancing system reliability. Furthermore, the antenna system demonstrates favorable radiation directivity, contributing to efficient signal transmission and reception within the designated frequency ranges. In summary, the antenna system meets functional requirements and exhibits reliability across various frequency bands, positioning it as a favorable choice within the 5G industry landscape and for experimental setups. Its compact size, wide frequency coverage, and reliable performance make it a promising candidate for integration into future 5G-enabled devices and infrastructure, thereby facilitating enhanced connectivity and communication capabilities.

## Figures and Tables

**Table 1:** Comparison of the 5G MIMO smartphone antennas.

Parameters	Value (mm)	Parameters	Value (mm)
L_1_	5.3	L_5_	1.5
L_2_	6.1	L_6_	12
L_3_	1.8	L_S_	10.5
L_4_	1.5	H_2_	3.4
H_3_	2.2	T_1_	2
H_4_	0.8	T_2_	12
H_5_	1.9	T_3_	6.2
H_6_	1.2	T_4_	2.3

**Table 2:** Performance contrast between the proposed miniature eight-port antenna array and other works.

Reference	Operating Band (GHz)	Isolation (dB)	Efficiency (%)	ECC	Size (mm^3^)	Gain (dB)
[6]	3.1–3.9 5.5–6.3	>12	51–84 49–72	<0.035	150 × 75 × 7	-
[7]	3.3–5.95	>15	47–78	<0.11	150 × 75 × 6	-
[9]	3.4–3.6	>20	33–47	<0.4	150 × 75 × 5.3	-
[18]	3.3–3.6	>15	47–65	<0.15	124 × 74 × 6	1.95–4.8
This work	3.4–3.6 4.7–5.3	>20	43–61 68–85	<0.066	150 × 75 × 5	1.86–5.32

## Data Availability

The original contributions presented in the study are included in the article, further inquiries can be directed to the corresponding authors.
